# Supplementary material for: Effect of COVID-19-Related Lockdown οn Hospital Admissions for Asthma and COPD Exacerbations: Associations with Air Pollution and Patient Characteristics
Source: J Pers Med. 2021 Aug 30;11(9):867. doi: 10.3390/jpm11090867 (PMC8465209; doi:10.3390/jpm11090867)
Supplement: Supplementary file 1 [file jpm-11-00867-s001.zip › jpm-1270217-supplementary.pdf]

Effect of COVID-19-related lockdown on hospital admissions for asthma and COPD exacerbations: associations with air pollution and patient characteristics

Ioanna Sigala , Timoleon Gianakas, Vassilis G. Giannakoulis, Efthimios Zervas, Aikaterini Brinia, Niki Gianiou, Andreas Asimakos, Efi Dima, Ioannis Kalomenidis and Paraskevi Katsaounou

Data Supplement

S1. Scatterplots of air pollutant levels and admissions per week (total, asthma and COPD) for the year 2020

(All air pollutants are measured in μg/m³ while CO is measured in mg/m³)

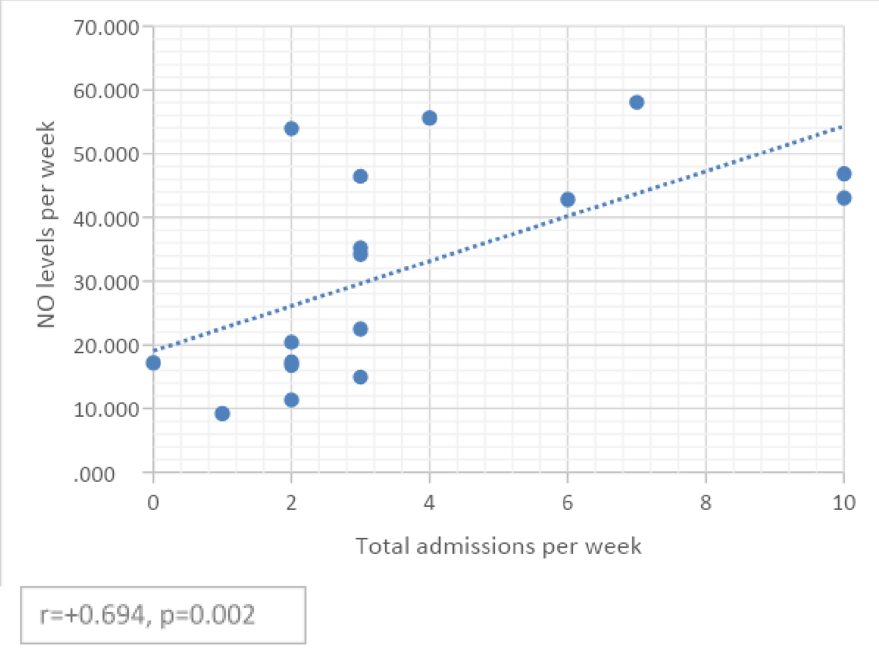

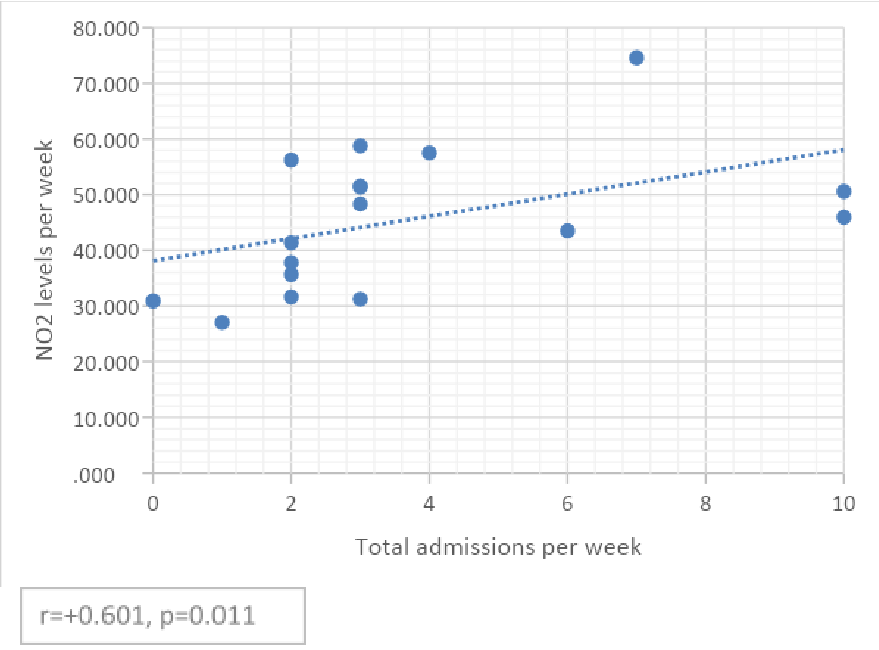

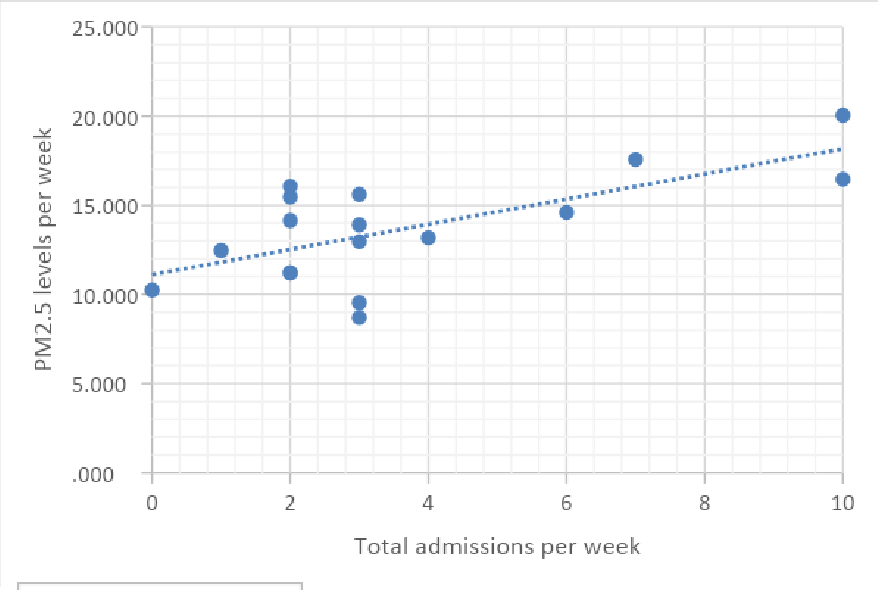

r=+0.526, p=0.030

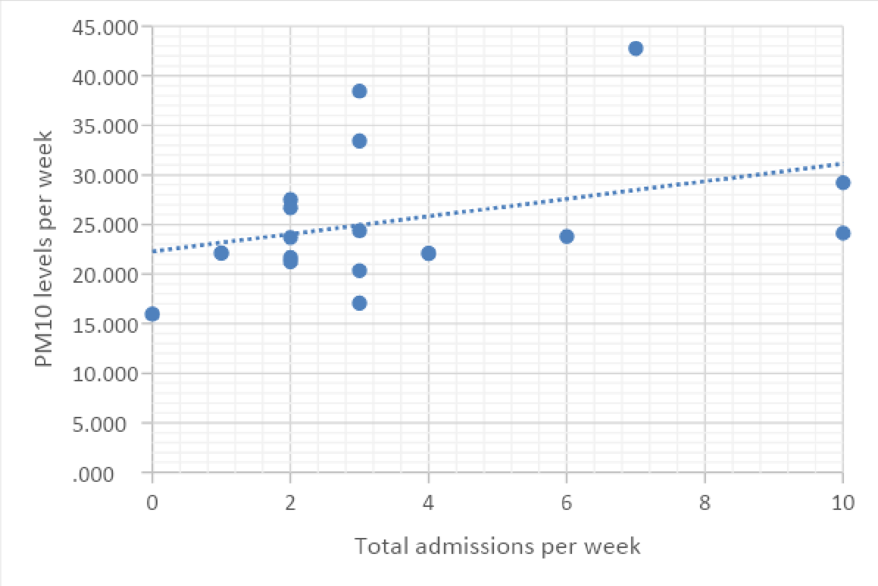

r=+0.435, p=0.081

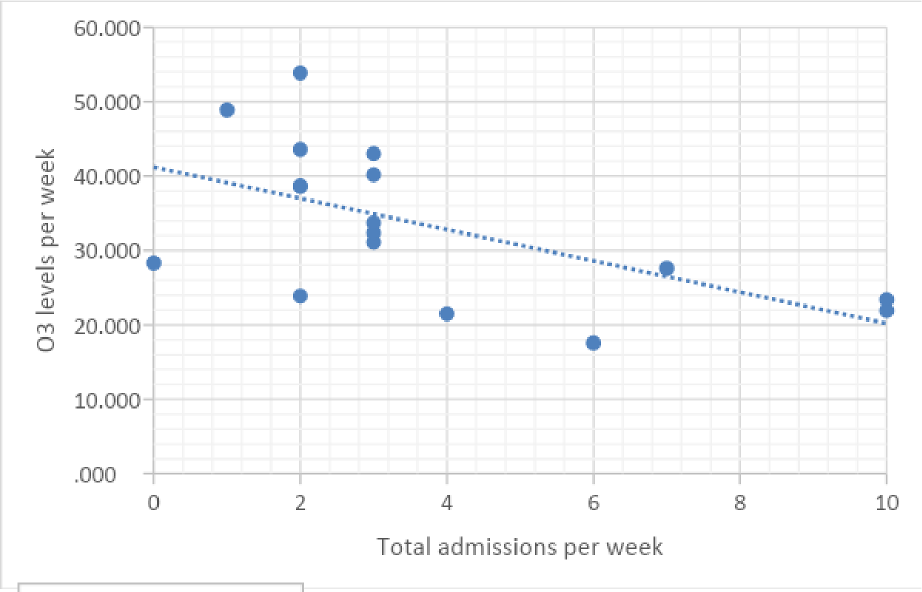

r=-0.635, p=0.006

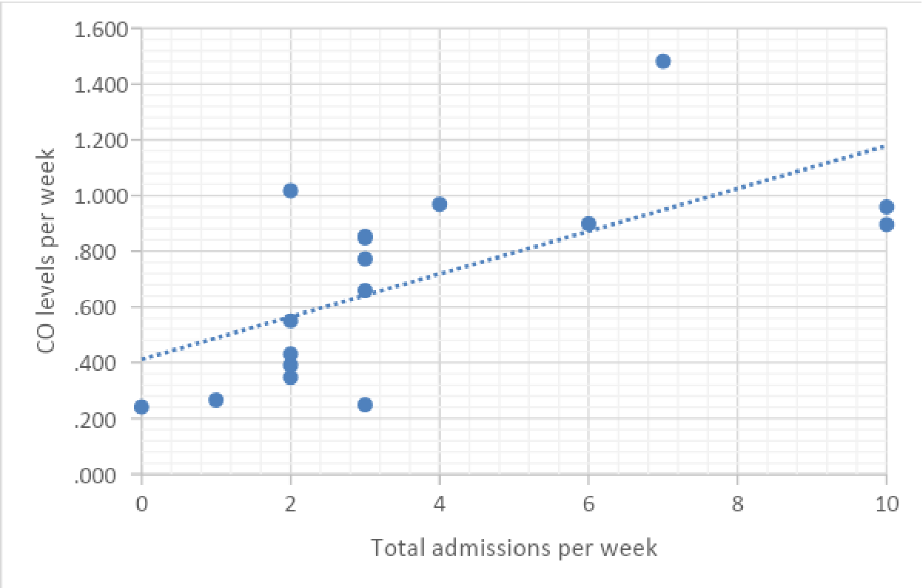

r=+0.707, p=0.002

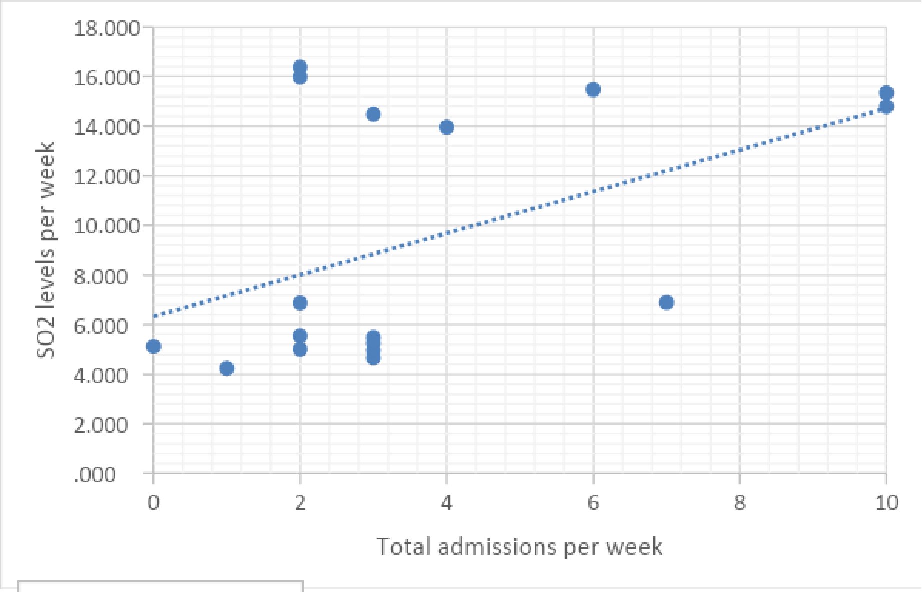

r=+0.373, p=0.140

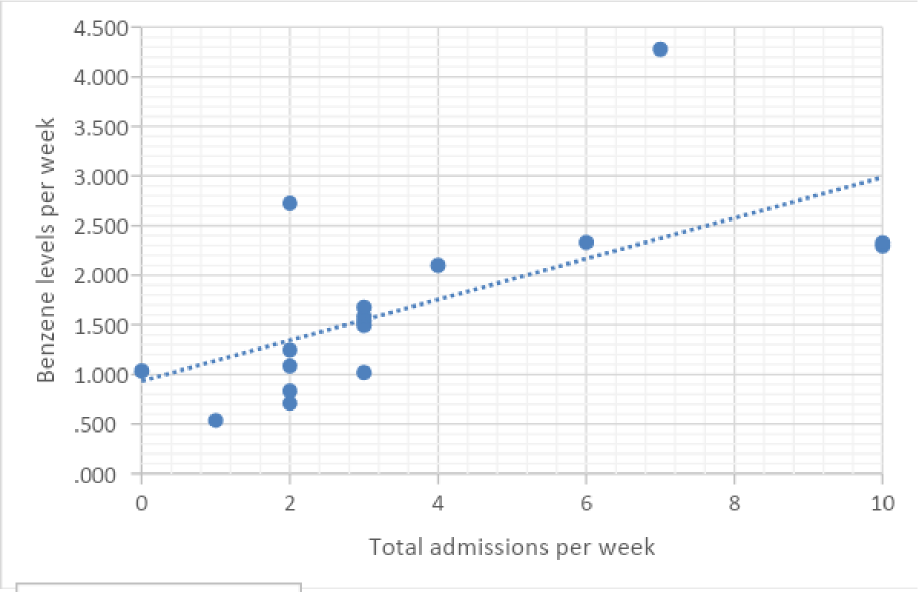

r=+0.721, p=0.001

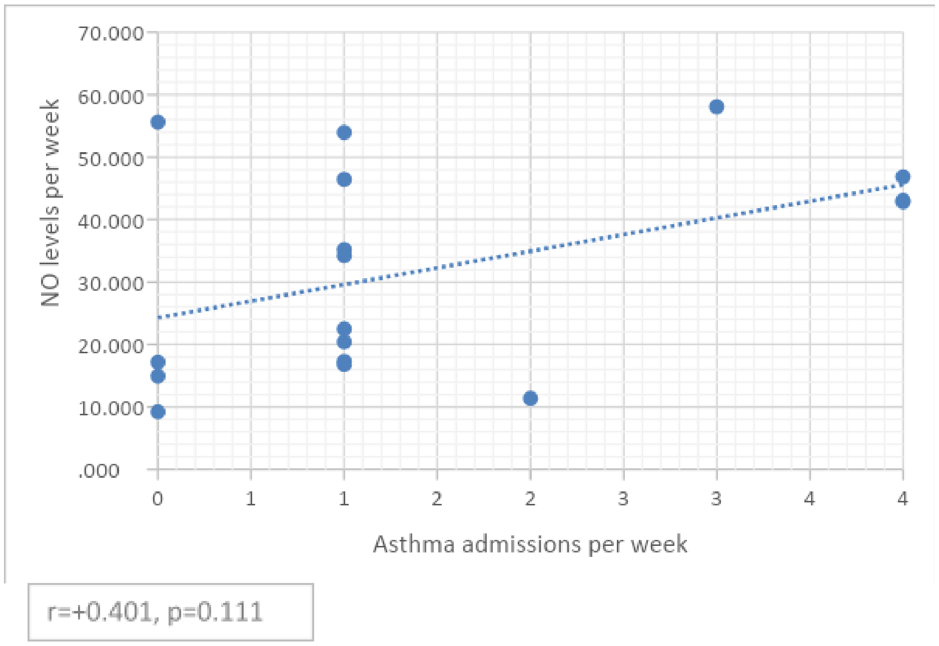

r=+0.401, p=0.111

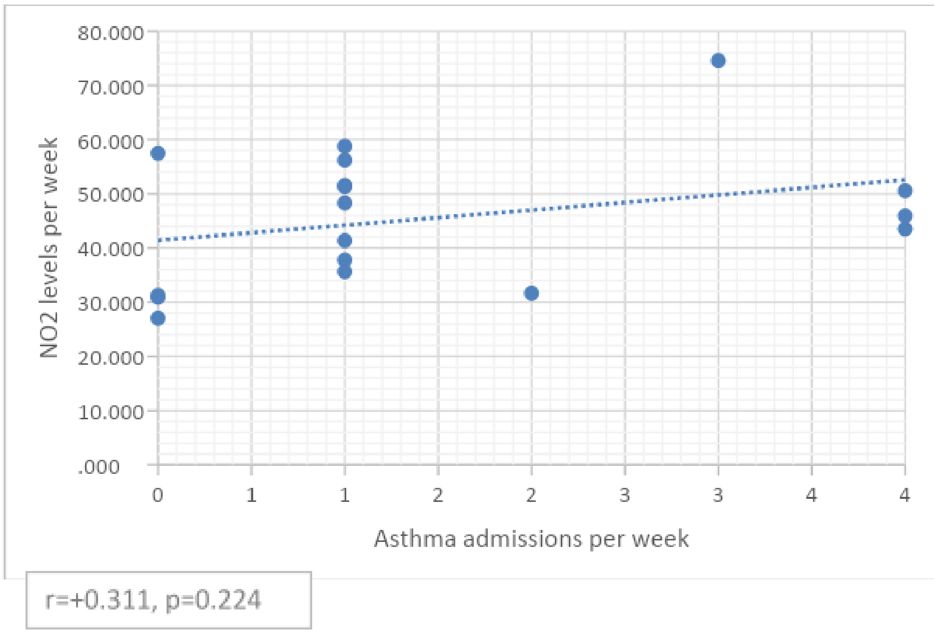

r=+0.311, p=0.224

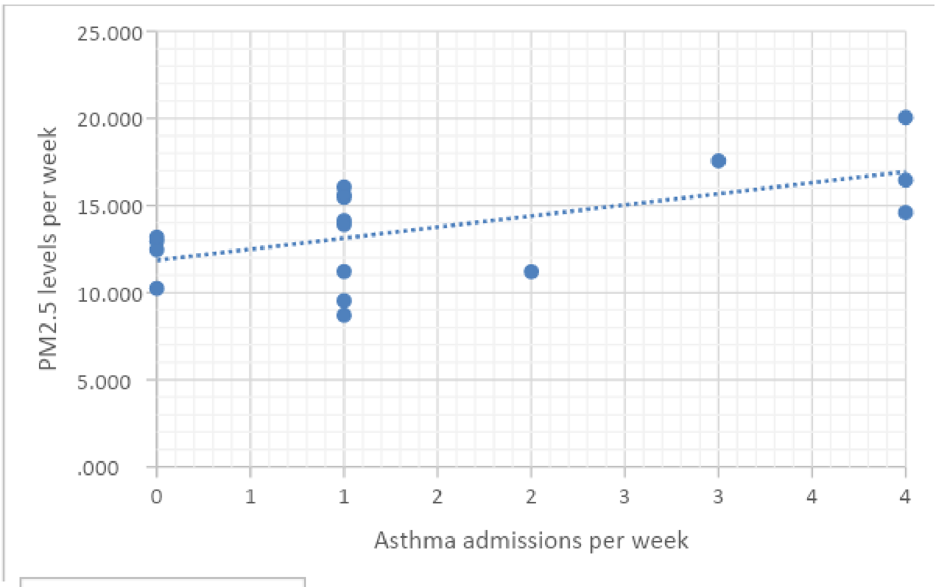

r=+0.543, p=0.024

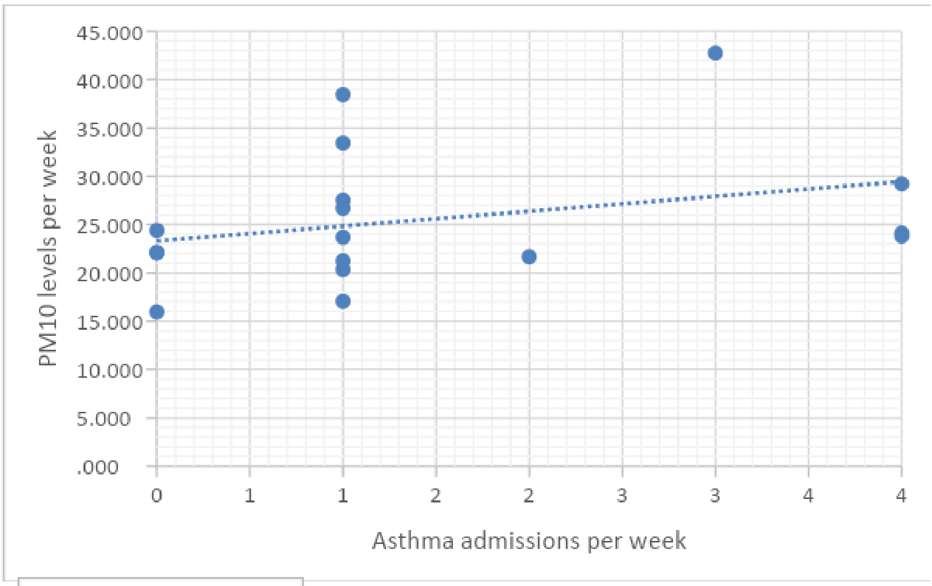

r=+0.358, p=0.158

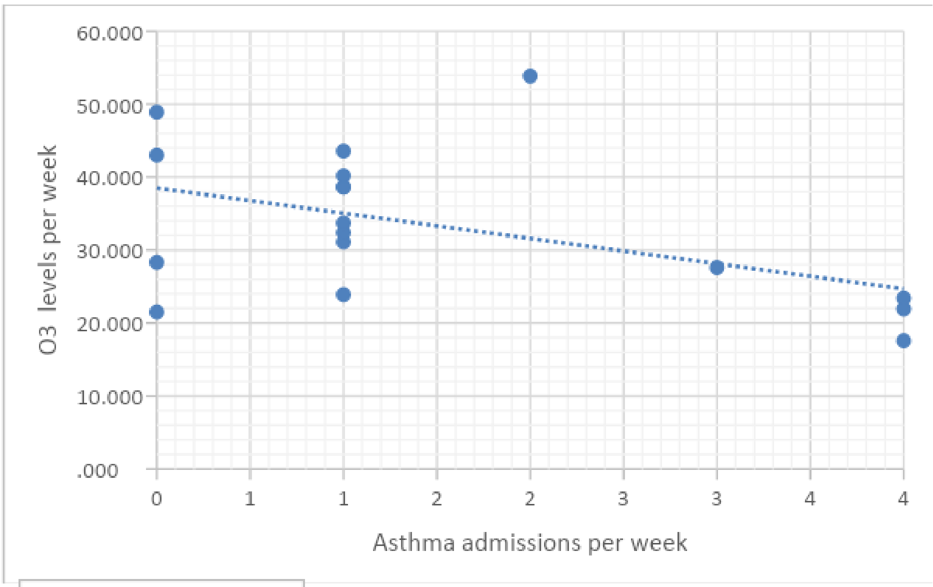

r=-0.368, p=0.146

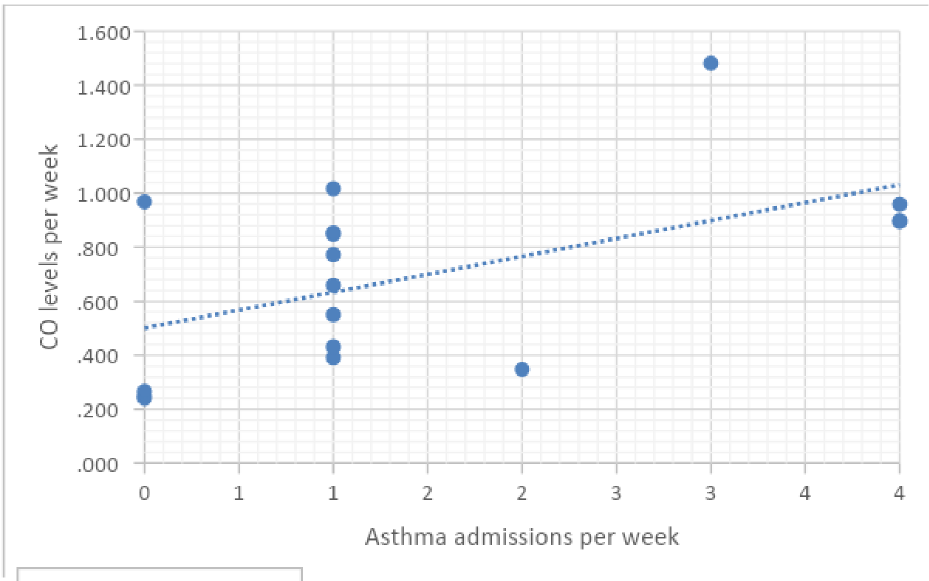

r=+0.526, p=0.030

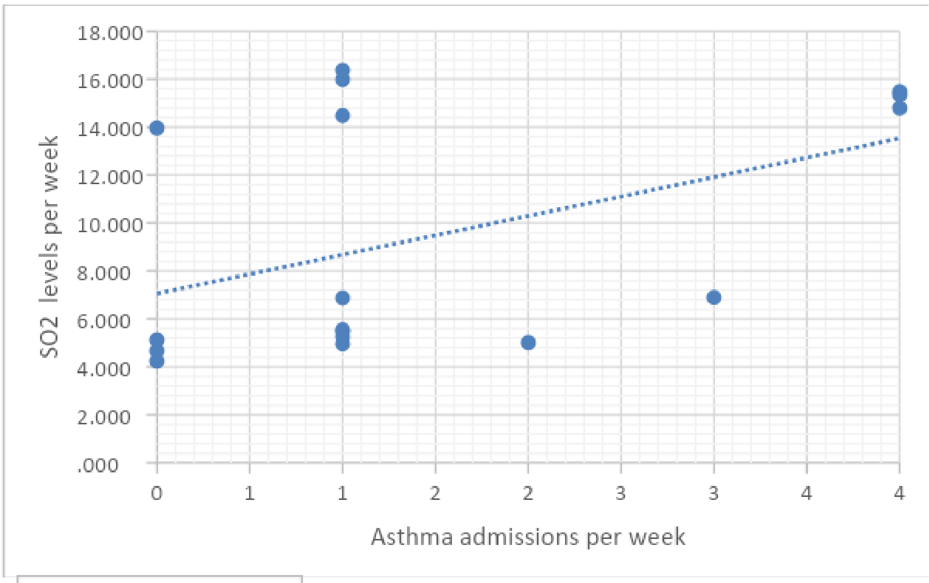

r=+0.516, p=0.034

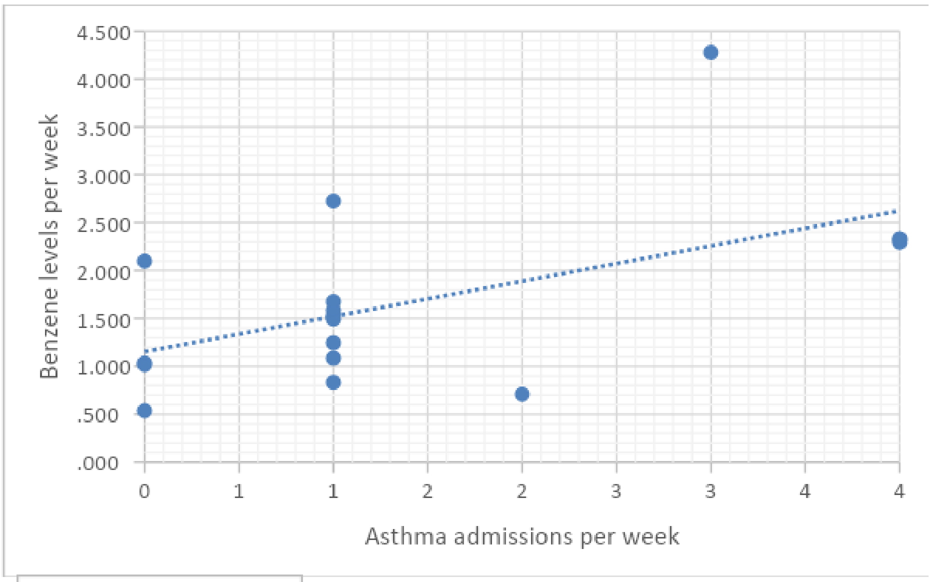

r=+0.546, p=0.023

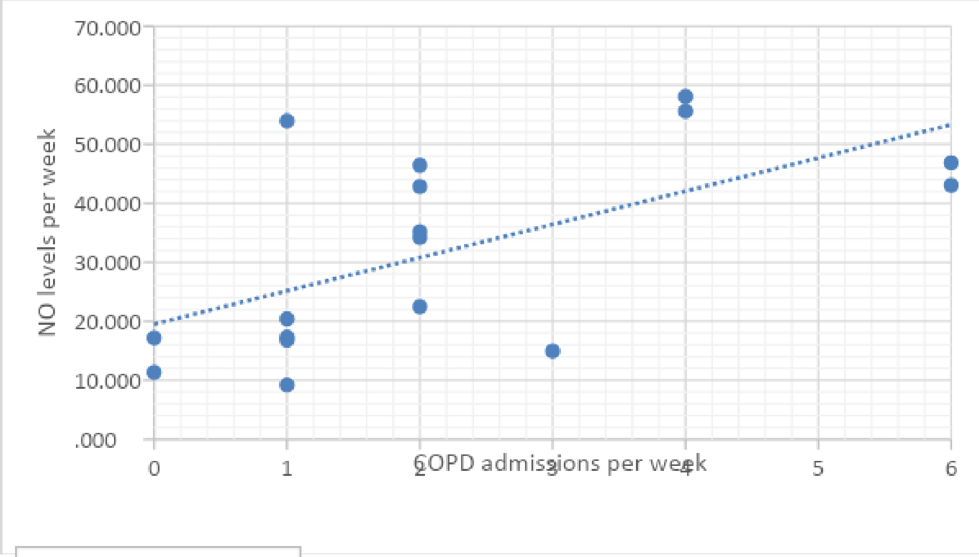

r=+0.642, p=0.005

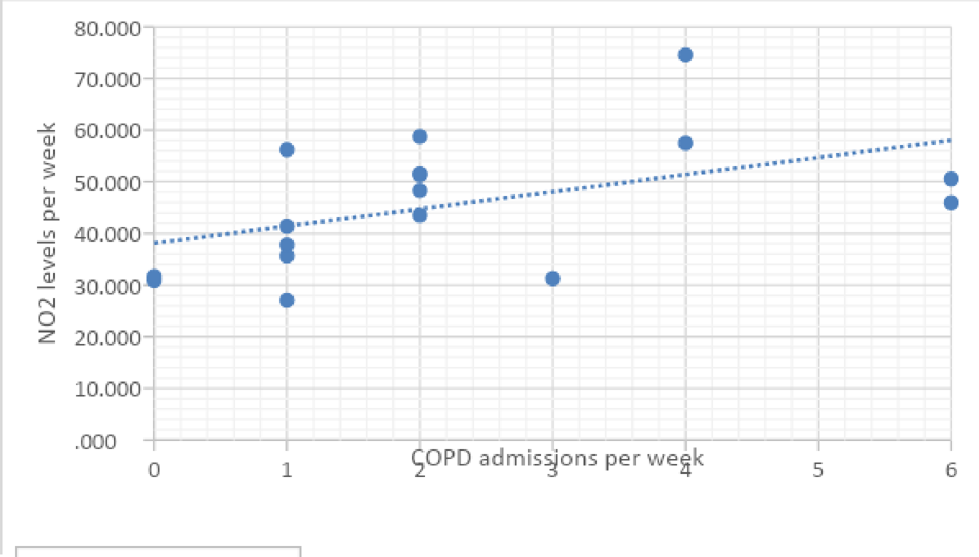

r=+0.554, p=0.021

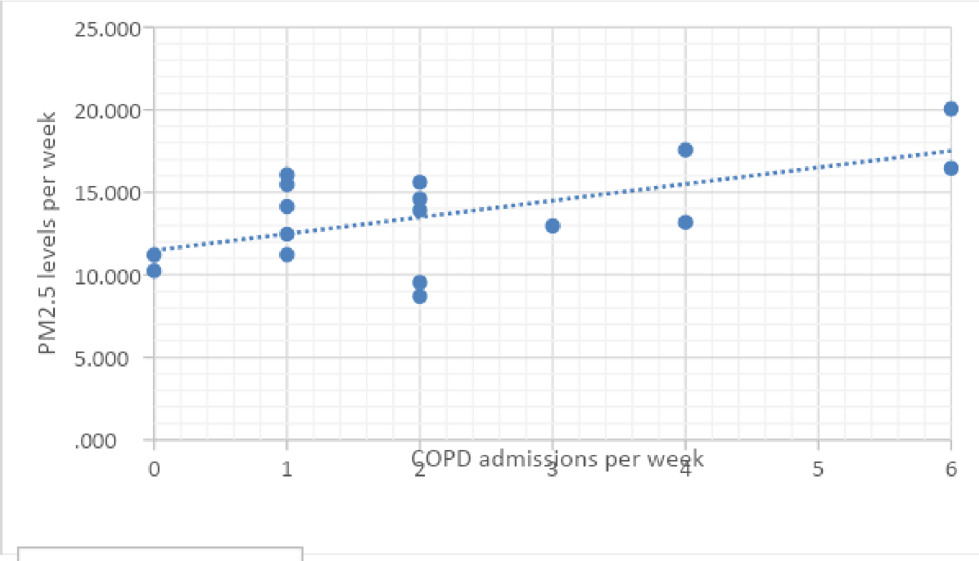

r=+0.492, p=0.045

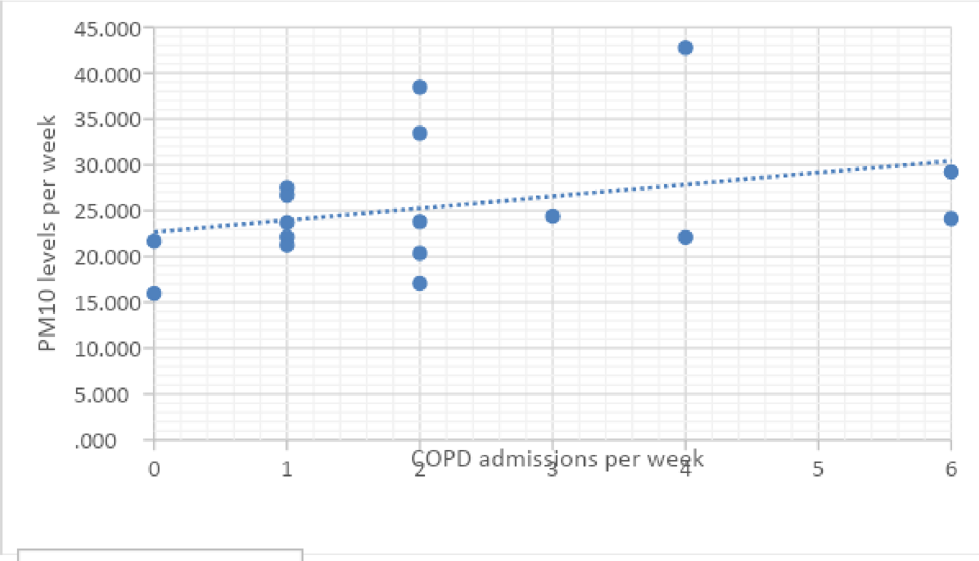

r=+0.439, p=0.078

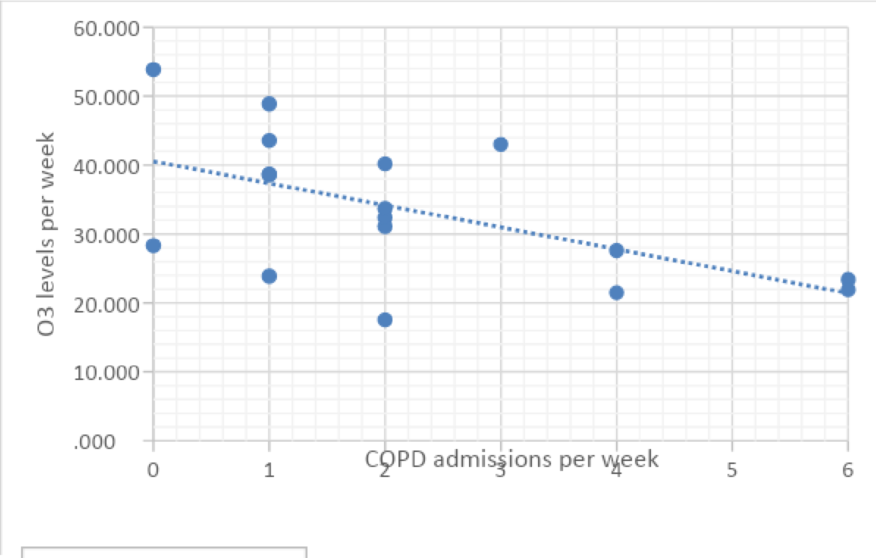

r=-0.56, p=0.019

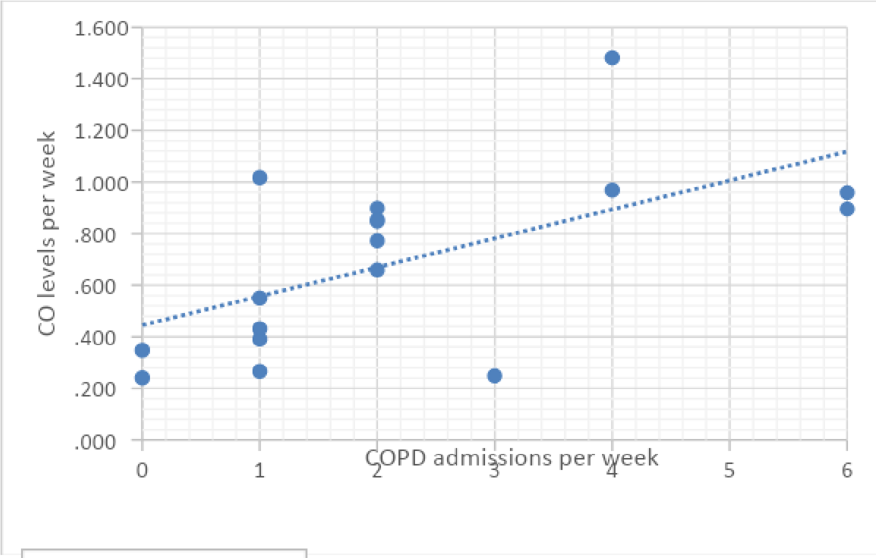

r=+0.616, p=0.009

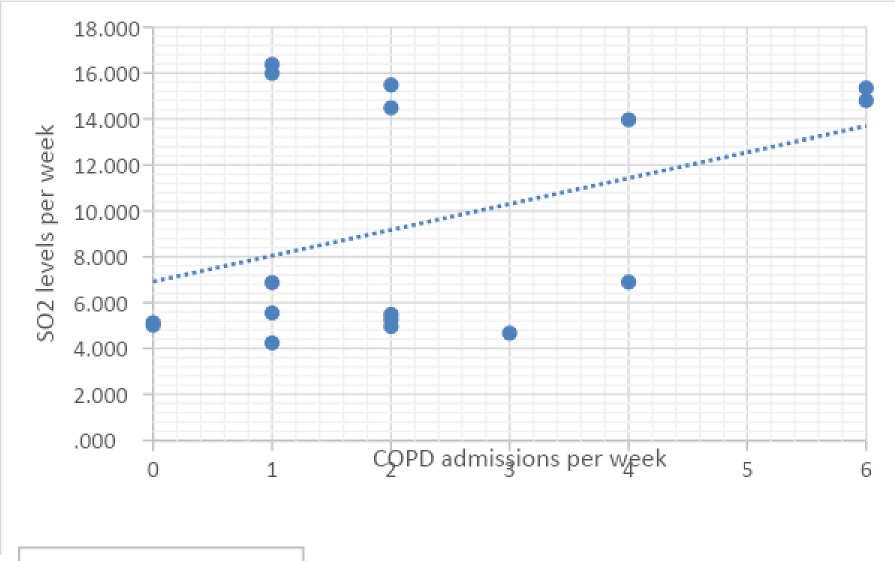

r=+0.246, p=0.342

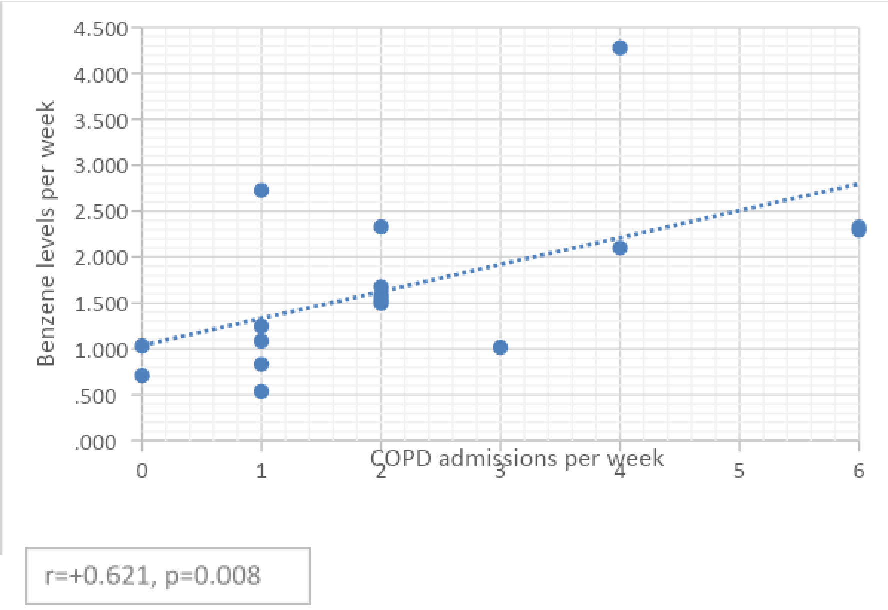

r=+0.621, p=0.008
